# A Place for Viruses on the Tree of Life

**DOI:** 10.3389/fmicb.2020.604048

**Published:** 2021-01-14

**Authors:** Hugh M. B. Harris, Colin Hill

**Affiliations:** ^1^APC Microbiome Ireland, College of Medicine and Health, University College Cork, Cork, Ireland; ^2^School of Microbiology, University College Cork, Cork, Ireland

**Keywords:** viruses, Tree of Life, evolution, phylogeny, horizontal gene transfer

## Abstract

Viruses are ubiquitous. They infect almost every species and are probably the most abundant biological entities on the planet, yet they are excluded from the Tree of Life (ToL). However, there can be no doubt that viruses play a significant role in evolution, the force that facilitates all life on Earth. Conceptually, viruses are regarded by many as non-living entities that hijack living cells in order to propagate. A strict separation between living and non-living entities places viruses far from the ToL, but this may be theoretically unsound. Advances in sequencing technology and comparative genomics have expanded our understanding of the evolutionary relationships between viruses and cellular organisms. Genomic and metagenomic data have revealed that co-evolution between viral and cellular genomes involves frequent horizontal gene transfer and the occasional co-option of novel functions over evolutionary time. From the giant, ameba-infecting marine viruses to the tiny Porcine circovirus harboring only two genes, viruses and their cellular hosts are ecologically and evolutionarily intertwined. When deciding how, if, and where viruses should be placed on the ToL, we should remember that the Tree functions best as a model of biological evolution on Earth, and it is important that models themselves evolve with our increasing understanding of biological systems.

“The very essence of the virus is its fundamental entanglement with the genetic and metabolic machinery of the host.”

Joshua Lederberg (American Nobel laureate, 1993)

## Introduction

Viruses infect all cellular life. Their evolution is inextricably bound to their target cells. Whether lyzing cells as part of a lytic cycle or inserting their DNA into the host genome in the lysogenic cycle, viruses place selective pressure on cells to evolve counter measures to evade infection. This, in turn, forces the virus to evolve further to avoid the defensive strategies of the host (Sinha et al., 2017). A dynamic and long-standing co-evolution stems from the ecological interactions of viruses with host cells. These interactions have traditionally been viewed as predatory and simply favor viral replication, but research into the effect of bacteriophages on microbial populations indicates that viruses may well be essential for ecosystem diversity (Braga et al., 2018).

Viruses lack a ribosome, preventing them from making their own proteins. Instead, they use the host cellular machinery to translate their messenger RNA (mRNA) into proteins, allowing them to assemble and multiply (Raoult and Forterre, 2008). Virus genomes are composed of either DNA or RNA and can be single-stranded (ss) or double-stranded (ds). They are divided into seven Baltimore classes, which also include positive-strand (+ss) and negative-strand (-ss) RNA viruses as well as two classes of retrovirus (Koonin et al., 2020a). Recently, a proposed megataxonomy of the virus world has placed viruses in a hierarchical taxonomic structure similar to that of cellular life (Koonin et al., 2020a). Almost all viruses encode capsid proteins that enclose and protect their genetic material; an exception to this is satellite viruses that rely on other viruses for their encapsidation (Brüssow, 2009).

Sequencing and analysis of viral genomes reveals that species phylogenies can be built in the same way as those of cellular genomes, despite the rapid mutation rates and regular gene exchanges between viruses (Gorbalenya and Lauber, 2017). This is because viruses are modular in their genetic structure, with structural and replication gene clusters often co-evolving separately as evolutionary units, while other less essential parts of the genome have been described as “flamboyantly mosaic,” meaning they are subject to frequent gene swapping (Hatfull and Hendrix, 2011). The accuracy of a phylogenetic tree of viruses therefore depends on whether the genes used are largely vertically inherited, or whether they move regularly between species. Unlike cellular organisms, viruses do not have genes that are common to all species and so a single viral phylogenetic tree cannot be created. This might not be possible even in principle because recent evidence suggests they are polyphyletic in origin (Krupovic et al., 2019).

If building a comprehensive phylogenetic tree of viruses is impossible, why should they be incorporated into the cellular Tree of Life (ToL)? After all, there are many who think that the questionable nature of their living status is enough to keep them excluded (Moreira and López-García, 2009). But “Life” has always been somewhat of a philosophical concept, open to counterexamples and logical inconsistencies (Cleland and Chyba, 2002). What is not in question is that viruses are evolving biological entities that share a long evolutionary history with cellular organisms. Appreciating the ToL as a model of the history of biological evolution on Earth, it is reasonable to ask if viruses should have a place within this model. Another way of posing this question is to ask if the existing ToL can ever truly make sense of evolutionary relationships without considering the role of viruses? Just as importantly, the ToL is rightly a dynamic concept, changing with new knowledge and insights (Mindell, 2013). Entire groups of organisms were discovered that dramatically altered the ToL topology, from the discovery of the archaea (Woese and Fox, 1977) to the more recent Candidate Phyla Radiation (CPR) and DPANN groups detected by analyzing metagenomic sequences (Castelle et al., 2018).

The standard ToL can be viewed as a two-dimensional, bifurcating species tree with a root representing the last universal common ancestor (LUCA). Diversity is usually plotted on the *x*-axis and time (or evolutionary rate) on the *y*-axis. The prevalence of horizontal gene transfer (HGT) in prokaryotes has already cast doubt on this simplistic model (Bapteste et al., 2005). It is important to remember that macroscopic lifeforms are the exception rather than the rule when we consider the number of species on this planet. The Open ToL is an online initiative to maintain a comprehensive, dynamic, and digital species ToL that, at its outset, included 2.3 million species (Hinchliff et al., 2015). We can only imagine how sophisticated and multi-dimensional such a digital model could be in principle, albeit not yet in practice.

The tide is turning regarding the role of viruses in the ToL (Forterre, 2005, 2006; Brüssow, 2009; Koonin et al., 2009b; Ludmir and Enquist, 2009). Genome sequencing and analysis of virus genomes give us unprecedented insight into their evolution and their relationships with cellular organisms (Hatfull and Hendrix, 2011; Dion et al., 2020). Metagenomics is now the primary means for identifying novel virus genomes. The crAss-like phage group—a dominant component of the human gut virome—and hundreds of novel ssRNA viruses have been discovered completely through metagenomic analyses (Callanan et al., 2020; Koonin and Yutin, 2020). The complexity of the evolutionary process is staggering, from the level of species to individual molecules.

This is a time for biologists to keep an open mind—there is still so much we do not know. Should a future ToL include viruses, or will they forever be kept apart from our models of cellular life?

## What Is Life and Does It Matter?

### The Struggle to Define Life

Are viruses alive? The question seems to be as much about Philosophy as Biology. A thought-provoking article from 2009 gives 10 reasons why viruses should not be included on the ToL and the first reason on their list is that viruses are not alive (Moreira and López-García, 2009). But is this true and does it even matter?

Modern definitions of life have lost the magical ways of thinking that haunted past generations: we no longer subscribe to the belief that fleas appear spontaneously from dust, maggots from meat or mice from mud. It was Louis Pasteur in 1859 who showed that even microorganisms do not originate from non-living matter and are only found growing on meat broth once the broth is first exposed to particles (bacteria) in the air (Berche, 2012). The “vital forces” that give rise to life have disappeared from our hypotheses, replaced by a materialistic approach that seeks to explain biological phenomena with concepts rooted in physics and chemistry. But life has yet to lose all of its mystery and an all-inclusive definition still seems to be beyond our grasp (Benner, 2010).

Erwin Schrodinger in his book of 1944—*What is Life?*—echoed the popular scientific view that all life is cellular, an assumption captured in the book’s subtitle: *The Physical Aspect of the Living Cell*. This view is still popular today and a cellular structure is a hallmark of living systems (Yewdall et al., 2018). Other common properties of life include metabolism, growth and development, homeostasis, reproduction, heredity, responsiveness, and evolution by natural selection (Madigan et al., 2018). There is no doubt that a biological entity with all these properties is considered “alive” and that one with none is “dead” or inert, but what about those that lie in between? Checklist definitions are useful, but they depend on how well the phenomenon of interest is understood. We want a combination of properties to be fully inclusive of true positives while also excluding false positive cases. Using logical terminology, the properties act as necessary and sufficient conditions for life. In other words, a set of properties must all be present and the presence of only these properties is enough to categorize an entity as living (Cleland, 2012). Unfortunately, there is no gold standard for “life,” no external vantage point from which we can evaluate the accuracy of a checklist of properties, even if the list itself allows us to think more clearly about biological organisms.

There are many other definitions of life. A definition by Gerald Joyce, pioneer of *in vitro* RNA evolution, is currently endorsed by NASA: “Life is a self-sustaining chemical system capable of Darwinian evolution.” Viruses are excluded since they lack the ability to self-sustain, needing host cells to replicate. Alternatively, a more inclusive definition of evolution by Richard Dawkins suggests a place for viruses in living systems: “Life results from the non-random survival of randomly varying replicators.” It has also been argued that “viruses neither replicate nor evolve, they are evolved by cells” (Moreira and López-García, 2009). This can be considered as simply semantics, but it highlights how difficult it can be for scientists to agree on the properties that viruses share with cellular organisms.

An insightful rebuttal to Moreira and López-García’s claim that viruses are evolved by the host cell is the concept of the virocell. Forterre explains that “the intracellular phase has been largely excluded from traditional virus definitions” and he differentiates between the metabolically inactive, extracellular state of a virus (the virion) and the metabolically active, intracellular state (the virocell). The virus hiijacks the cellular machinery of the host (the ribocell) to effectively become a living, cellular organism that produces a large number of virions instead of reproducing by cellular division. This manipulation of the host cell for the benefit of the virus is especially clear in cases where the host genome is completely inactivated or destroyed prior to virion production (Forterre, 2016). According to the virocell concept, if a cell is a living entity then so too is a virus, at least when its genes are being translated into proteins within an infected host. All life can still be cellular under this view, but there are competing strategies within the cell for replication, depending on the genome that is in control of the ribosome.

The conclusion that viruses are not alive is premature. The same can be said about statements on their living nature. Carol Cleland stresses that each definition of life encounters problems, quite often in the form of a robust counterexample (Cleland and Chyba, 2002). For instance, endoparasitic bacteria are also dependent on a host cell to survive, but their status as living is rarely questioned (Brüssow, 2009), even though their lack of self-sufficiency places them outside of NASA’s accepted definition of life. A similar argument has been made for the non-living nature of mitochondria and chloroplasts. These organelles are the descendants of free-living bacteria and likely only differ from bacterial endoparasites by the length of evolutionary time they have been dependent on a host cell to survive. In this scenario, mitochondria were once living but are considered non-living organelles by many scientists today. Mitochondria lacking DNA altogether (mitosomes) further emphasize the continuum that seems to exist between living and non-living entities (Forterre, 2016). This conclusion is supported by Benner (2010) who agrees that there is no satisfactory definition or working theory of life that can be used to place all entities in existence into one of two categories, living or non-living.

### Asking the Right Question

Scientists who worked on the 1976 Viking mission to Mars had to grapple with the complex problem of detecting life from afar. The mission focused on finding microbial metabolism in the Martian soil. Radioactively labeled Carbon-14 (^14^C) compounds were added to the soil and this carbon isotope was later detected in gaseous form as ^14^CO_2_. One interpretation of this result is that resident microbes on Mars metabolized the ^14^C compounds to produce gases. But there is still no consensus on whether microbial metabolism was detected on Mars (Levin and Straat, 2016). The Viking mission focused on detecting Earth-like metabolic signals, but we can speculate that lifeforms in other galaxies may be too strange to be found by our existing technologies or mindsets.

The discontinuity between life and non-life might not ultimately matter because we do not yet know (and may never know) if life is a natural category defined by the universe or an artificial one created by man (Cleland, 2012). Perhaps life-like entities throughout the universe do not share a common property. Conversely, life might always exhibit certain characteristics like evolution by natural selection. The concept of life is a human invention and might not accurately reflect the underlying reality we are trying to explain. Where does this leave us with viruses and the ToL? The “Life” part of the ToL may not be as important as what the ToL was designed to represent. It was never meant to be an exhaustive category of all living things, but more of a model of biological evolution on our planet (Doolittle and Brunet, 2016).

Like all models the ToL can (and should) change with new information. It is repeatedly modified and updated as new data become available. Mindell described it as a “metaphor, model, and research tool to explore life’s evolution and genealogical relationships.” He stressed that the ToL model has not become obsolete with the discovery of widespread HGT because it already has a “long history of adapting to incorporate new knowledge” (Mindell, 2013). Our elusive concept of life suggests that we should look to the dynamic nature of the ToL itself when asking if it has a place for viruses. The relevance of this question is reinforced by a recent publication by Koonin et al. (2020a) proposing a megataxonomy of the virus world. With the recent explosion of virus genomics and the accompanying interest in viral evolutionary history, is it only a matter of time before the ToL must accommodate viruses?

## The Tree of Life as a Dynamic Model

### Darwin and Company

**FIGURE 1:**
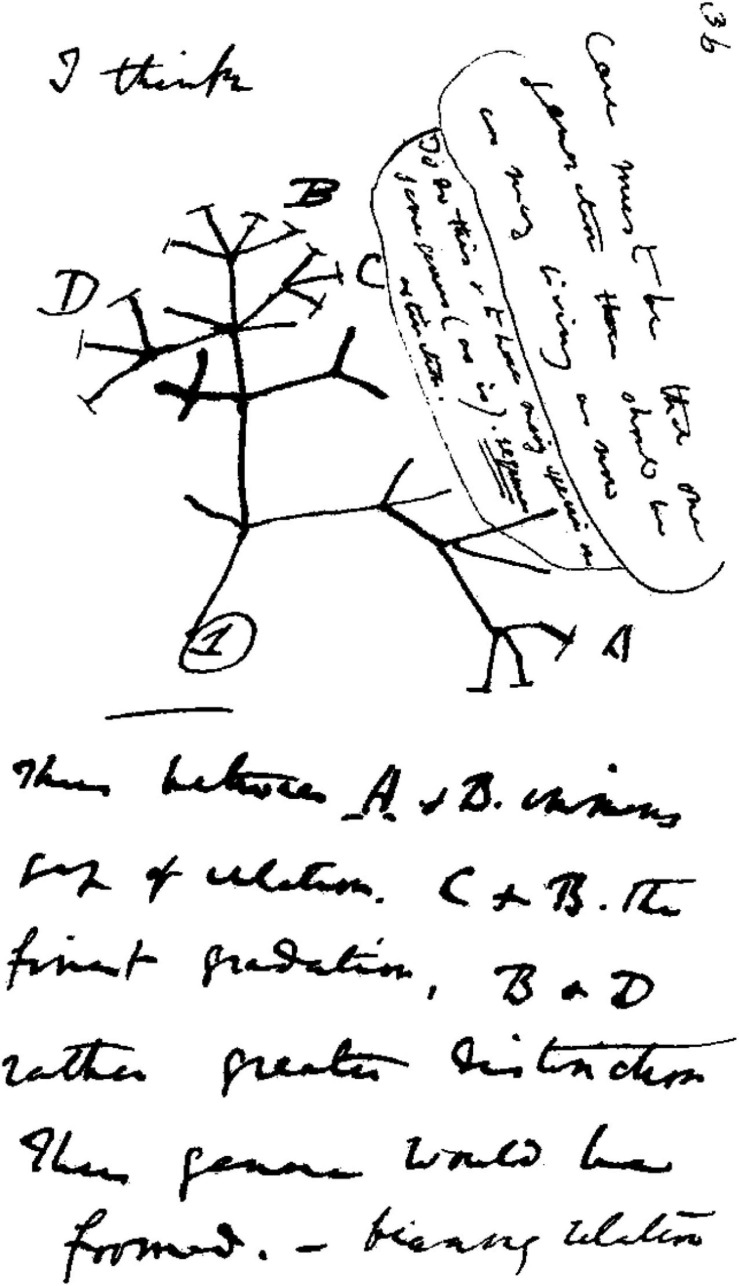
Charles Darwin’s 1837 sketch. His first diagram of an evolutionary tree from his First Notebook on Transmutation of Species (1837). Interpretation of handwriting: “I think case must be that one generation should have as many living as now. To do this and to have as many species in same genus (as is) requires extinction. Thus between A + B the immense gap of relation. C + B the finest gradation. B + D rather greater distinction. Thus genera would be formed.” Attribution: Charles Darwin/Public domain.

I think. These were the words written by Charles Darwin above a crude sketch of a tree from one of his notebooks in 1837 (Figure 1). By the time his scientific masterpiece, *On the Origin of Species*, was published in 1859 he had done a lot more thinking. This was the basis for the theory of evolution by natural selection. Darwin gave biologists a conceptual framework into which every species on the planet could intelligibly fit. The concept of evolution did not originate with Darwin, but he did propose the only plausible mechanism of how a species might change over time. He explained that variation in morphology, physiology, or behavior across organisms arises naturally and randomly. The environment (both biotic and abiotic) then selects those variants that best allow organisms to survive and reproduce (Darwin, 1859). Over geological time, he claimed, this led to all the diversity of life that we see today, from the actions of the humble earthworm to the complexity of the human eye.

Darwin visualized evolution as a tree on which two diverging branches represent the creation of two related but distinct species from a common ancestor. The process of speciation itself was hypothesized to occur when subpopulations of the same species became geographically isolated, gradually diverging over time until reproduction between separated organisms becomes impossible (Darwin, 1859). Darwin was speculating mainly on macroscopic species, but it is interesting to note that Pasteur famously refuted the spontaneous generation of microbes in the same year (Berche, 2012). Darwin also speculated that the diversity of species on the planet today arose from only a few primitive organisms, or perhaps only one: the great trunk of the ToL (Darwin, 1859).

Trees depicting biological relatedness existed before Darwin, but the assumed mechanism of speciation was different. Edward Hitchcock and Jean-Baptiste Lamarck both constructed trees showing the relationships across groups of species. Hitchcock, however, did not support evolutionary thinking and believed in separate acts of creation by God for each species. Lamarck supported evolutionary concepts but emphasized the inheritance by offspring of characteristics acquired during the lifetime of the parent organisms (Burkhardt, 2013). Deities played no part in Darwin’s theory, but he did support some of Lamarck’s ideas (Kováč, 2019).

**FIGURE 2:**
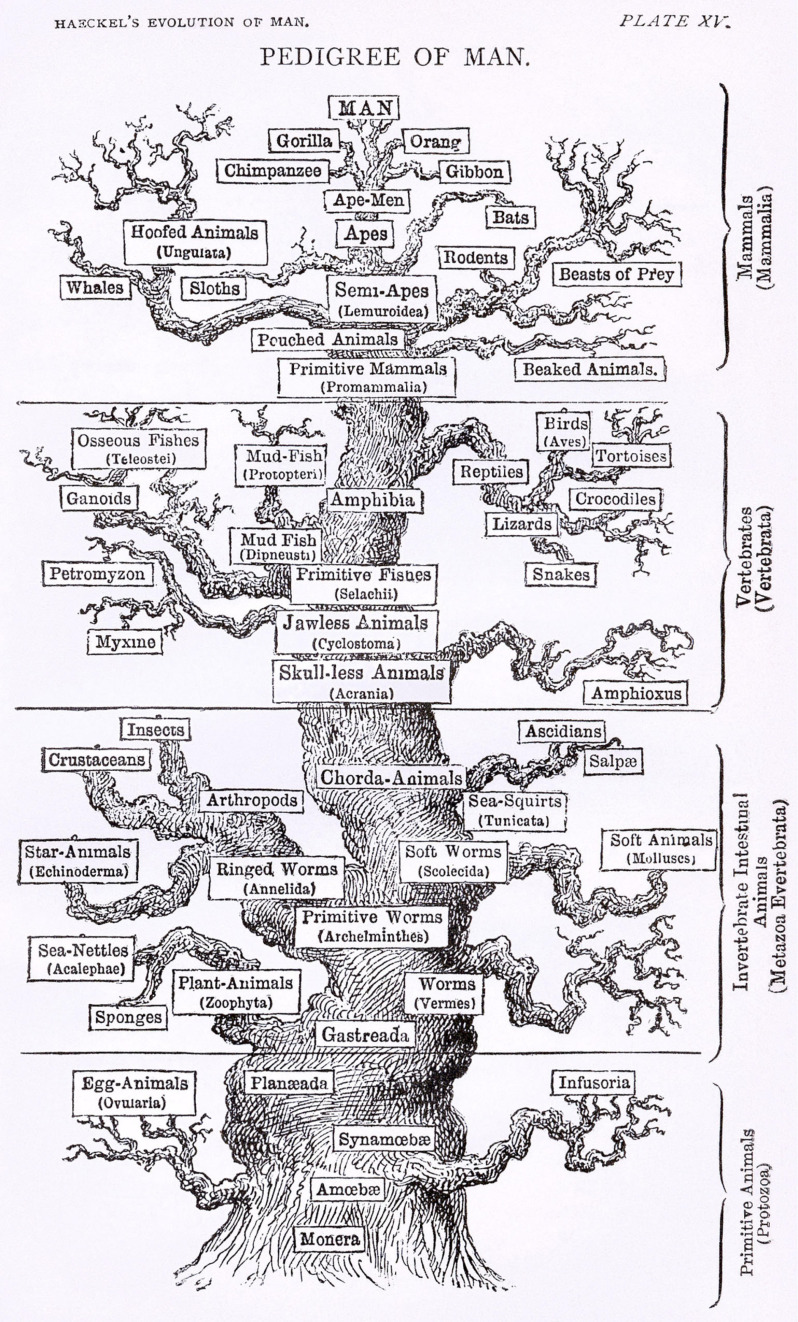
A version of the “tree of life” by Ernst Haeckel, from The Evolution of Man (1879). Attribution: Ernst Haeckel/Public domain.

Ernst Haeckel, a prominent zoologist and a contemporary, promoted Darwin’s work in Germany. Haeckel constructed several biological trees during his lifetime, far more detailed than Darwin ever had (Figure 2). Ironically, he favored Lamarckism over Darwinian natural selection, so while he popularized Darwin’s ideas he disagreed with his explanation of the mechanism of evolution (Watts et al., 2019). Haeckel also coined the term *phylogeny*, referring to tree-like patterns of biological evolution whose diagrammatic representations became known as phylogenetic trees (Levit and Hossfeld, 2019). These early trees were based on morphological comparisons: species with similar morphologies or a shared set of morphological characteristics were placed close together on the tree. Evolution from a common ancestor was therefore inferred from morphological similarity (Mindell, 2013).

Initial trees of life reflected an underlying evolutionary reality, but their accuracy depended on the assumption that a greater similarity in a chosen set of morphological characteristics is equivalent to evolutionary relatedness. This is not always the case. A well-known example of how morphology can disguise true evolutionary relationships is that whales and hippopotami are each other’s closest living relatives (Geisler and Theodor, 2009). This is even more true in the microbial world, where individual microbes can be very functionally and phylogenetically diverse yet share similar cellular morphologies (Woese et al., 1990). So, how do we know that whales and hippopotami are so closely related when it is not apparent from their morphology? This knowledge stems from robust independent phylogenetic methods, beginning with the sequencing and molecular comparison of genes and genomes.

### Carl Woese and His Favorite Gene

Carl Woese wrote that “[a]n organism’s genome seems to be the ultimate record of its evolutionary history” (Woese and Fox, 1977). Woese pioneered the building of phylogenetic trees using molecular sequences of the 16S ribosomal RNA gene, an essential component of the prokaryotic ribosome (with a homologous form, 18S RNA, in eukaryotes). This was possible because sequences of the same gene in different species mutate and diverge over time. The evolutionary rate of molecular change is highly variable across species (Kuo and Ochman, 2009), in different genes (McInerney, 2006), and in different sites along a single gene (Echave et al., 2016). However, by comparing sequence similarity across species, it is possible to estimate the branching order of speciation events (i.e., their phylogeny).

The 16S rRNA gene has nine hypervariable regions separated by highly conserved stretches of DNA, which is transcribed into structurally constrained RNA. It is therefore subject to both neutral and purifying selection, where hypervariable regions diverge with increasing evolutionary distance while conserved regions essential for structure and function remain unchanged (Chakravorty et al., 2007). The 16S rRNA gene is also considered to be a reliable molecular clock because it is functionally constrained across diverse species, although the reliability of molecular clocks has come under scrutiny in recent years (Kuo and Ochman, 2009).

Carl Woese used the 16S rRNA gene to discover the third major domain of life, the archaea. Although methanogens were well studied at the time, they were thought to be a group of bacteria; Woese showed that, based on 16S sequences, they were an entirely separate group of organisms (Woese and Fox, 1977). In building a phylogeny of life from a single gene, Woese was making a tacit assumption: the evolutionary history of the gene represents the evolutionary history of species (Woese and Fox, 1977). It was a safe assumption at the time since genes reside on genomes that divide in synchronization with the cells they lie inside, and the same logic can be applied to individuals (multicellular), populations, and species. This all changed, however, with the comparison of whole genomes.

### A Disagreement Among Genes

Orthologous genes in different organisms originate from a common ancestral gene. We might naively assume that phylogenetic trees built from each orthologous gene across several species would share the same topology, a topology that ultimately reflects the evolutionary history of the species. This turned out to be false, particularly for prokaryotic genes. As more and more genomes were sequenced, two things became clear. The first was that not every strain of the same species shares the same set of genes (Tettelin et al., 2005). The second was that, even of those genes that are present in all strains of a given species, individual gene tree topologies differ (Galtier and Daubin, 2008). Incongruent gene trees meant that individual genes followed separate lines of descent through evolutionary time; in other words, genes sometimes jumped from one species to another. It became obvious that genome evolution was not as straightforward as scientists previously imagined.

The concept of the pangenome emerged from comparing bacterial genomes. The genomes of most species show a high degree of plasticity and genes are regularly gained and lost over time. This leads to a core genome that consists of genes shared by every strain of a given species, a dispensable genome shared by some strains and unique genes that are strain specific (Medini et al., 2005). Gene loss occurs through several different types of deletion mutations, while gene gain occurs through HGT, a phenomenon that is only recently being fully appreciated for its role in genome evolution. Evidence for the occurrence of HGT existed as far back as 1928 when the bacterial transformation of DNA was discovered (Petsko, 2006). It was only through the comparison of individual gene trees, however, that the widespread nature of HGT became obvious to the scientific community (Mindell, 2013).

The horizontal transfer of genes from one cell to another occurs by three mechanisms: transformation, conjugation, and transduction (Thomas and Nielsen, 2005). Transformation is the uptake of naked DNA from the environment by a cell, which can then be broken down into individual nucleotides or used in DNA repair (Lerminiaux and Cameron, 2018). Conjugation occurs between cells in contact when a bridge known as a pilus is formed that allows the transfer of DNA, usually by a plasmid. Transduction happens when a virus accidentally packages host DNA into its capsid, sometimes transferring this DNA to a new host. All three mechanisms allow new genes to be transferred from one species to another, potentially increasing the functional repertoire of genomes (Chiang et al., 2019). A fourth mechanism, vesiduction, has recently been proposed to explain the transfer of DNA by extracellular vesicles (EVs)—an event that has been observed in all three domains of life (Soler and Forterre, 2020). Another important player in HGT is the transposable element, a ubiquitous and very diverse group of genes that code for enzymes involved in splicing and insertion of their own DNA (Arinkin et al., 2019). Sequences that can be horizontally transferred are generally referred to as mobile genetic elements (MGEs).

Horizontal gene transfer forces us to reconsider the initial simplistic view of the ToL. If different orthologous gene trees have incongruent topologies (suggesting separate evolutionary histories), which one is correct? It has become obvious that some genes undergo HGT more than others. It is estimated that informational genes, mainly those involved in transcription and translation, are rarely transferred horizontally, and are constrained to strict lines of vertical inheritance. Alternatively, operational or “housekeeping” genes undergo HGT a lot more frequently. One popular explanation for this is the complexity hypothesis: it posits that because informational genes act together in complexes, they co-evolve as evolutionary units while many operational genes do not, and are therefore not penalized by selection in the same way as a result of engaging in HGT (Jain et al., 1999). This suggests that we should focus on informational genes if we want an accurate species ToL. After all, they seem to follow the same vertical lines of evolutionary descent as the species they represent. But there is also evidence of HGT in informational genes, and so no single gene can ever be used to guarantee an infallible species ToL (Galtier and Daubin, 2008).

Two illuminating concepts that broaden the range of possibilities are the phylogenetic forest (Koonin et al., 2009b) and the Statistical ToL (SToL) (Puigbò et al., 2013). The evolutionary history of each gene is depicted as a separate tree in a Forest of Life (FoL). Koonin et al. write that “[t]he totality of gene trees comprising the FoL appears to be a natural representation of the history of life given the inherent tree-like character of the replication process.” Here, the species ToL has been abandoned in favor of a more insightful interpretation of evolutionary history as the individual phylogenies of all gene trees. Should the notion of a single ToL be replaced? Puigbo et al. took 6,901 phylogenetic trees for prokaryotic genes, identifying a significant central trend representing a signal of vertical inheritance. This signal was particularly strong in a subset of 102 nearly universal trees (NUTS), which include genes involved in transcription and translation. While the central trend cannot replace all gene trees in the FoL, which are highly incongruent, it does give us an SToL that acts as a conceptual backbone within which HGT takes place (Puigbò et al., 2013).

**FIGURE 3:**
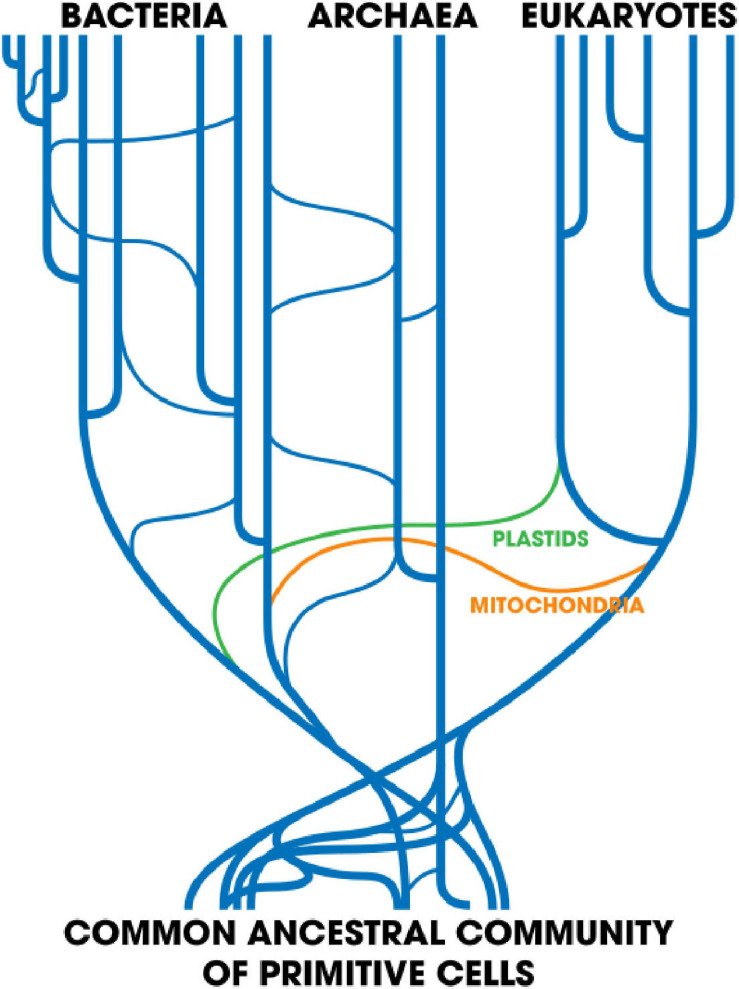
Taken from Smets and Barkay (2005). Horizontal gene transfer: Perspectives at a crossroads of scientific disciplines. Created: 23 May 2018; License: CC-BY-SA 4.0.

Another interesting concept is that of a network of life (Ragan et al., 2009). It can be visualized as a tree (the vertical component) with instances of HGT represented by crisscrossing lines linking distant branches (the horizontal component) (Figure 3). Models like this are no doubt more accurate at reflecting the real history of biological evolution. The most familiar case, perhaps, is the acquisition of free-living cellular ancestors of mitochondria and chloroplasts by a primitive eukaryote (López-García and Moreira, 2015). Far more comprehensive networks involving many separate HGT events might be more reflective of the underlying evolutionary reality.

The very concept of discrete species disappears under these views. They take on a more malleable nature as phenotypic expressions of collections of genes, where genes occasionally move from one collection to another. In, *The Selfish Gene*, Richard Dawkins refers to organisms as “survival machines” and stresses the importance of a gene’s eye view of evolution. Dawkins saw each gene as trying to maximize its own success in terms of the number of copies existing in the world. A genome can be thought of as a team of genes with the same goal and each gene contributes to the phenotypic expression of the genome (via proteins in the case of coding genes). Success or failure of the phenotype is therefore determined by selection acting on each gene in the context of its environment (including other genes) (Dawkins, 1976). Genes that can transfer themselves horizontally as well as vertically (via cell division) can be viewed as breaking away from this collaborative enterprise, although the causes behind the origin and evolution of HGT are no doubt manifold, some of which, perhaps, have yet to be discovered.

Theoretical models predict the evolution of cooperation in simple molecular replicators (Levin and West, 2017). They also predict the inevitable emergence of parasitic behavior from cooperative systems (Koonin et al., 2017). This suggests that once genes started acting in concert to build a cell and its associated metabolism, the evolution of rogue genes that mutated to exploit their neighbors was unavoidable. Many forms of HGT, including viral infection, might in principle be explained by this reasoning, although the creation of genomes from individual genes most likely involved forms of HGT that allowed for sequence splicing and insertion (Gilbert, 1986). There was a time before genes cooperated, existing instead as individual replicator molecules. According to the RNA world hypothesis, these replicators were molecules of ribonucleic acid with limited catalytic ability (Gilbert, 1986). The need to continuously produce energized nucleotides makes it more likely that they were compartmentalized, perhaps in lipid vesicles, and had a primitive proto metabolism (Forterre, 2005). The RNA world is still controversial, but it remains one of the most plausible explanations for the early stages of the evolution of life (Bernhardt, 2012). But what about the first genomes? More specifically, what about the common ancestral genome of all cellular life? This is the root of the ToL, representing the population of cells whose countless divisions led to every biological cell in existence today.

### Is LUCA Lost in Time?

The acronym LUCA has been used to either stand for the LUCA or the last universal cellular ancestor. While the scientific consensus is to use LUCA to refer to the common ancestor of all modern cells, the distinction is important because the common ancestor and the cellular ancestor can logically be different. For example, if viruses evolved from ancient cells, they would predate the last universal cellular ancestor, but not necessarily the LUCA (Nasir et al., 2012). Alternatively, the LUCA might have been acellular. In a sense, the LUCA depends on the biological entities we are considering, while the last universal cellular ancestor does not, because it is defined by cellular life existing across three domains. Unless one or more of Bacteria, Archaea or Eukarya goes entirely extinct, the last universal cellular ancestor will remain the same. LUCA as the common ancestor of all modern cells is how the acronym is treated for the rest of this review. A related concept is the first universal common ancestor (FUCA), which is the common ancestor of all modern cells as well as ancient cellular lineages that are now extinct.

The dynamic nature of the ToL reached all the way back to its root. An organism so ancient will never be properly defined, but recent comparative genomic research is unveiling some of the mystery surrounding LUCA. Detailed analyses of the multitude of genes spread across all extant lifeforms reveal likely genetic, metabolic, and environmental traits (Mat et al., 2008). One study hypothesized that LUCA is a thermophilic, single-celled organism that inhabited hydrothermal vents and had a complex and functionally diverse gene repertoire (Weiss et al., 2016). This interpretation has come under criticism from Berkemer and McGlynn who show with improved sampling of homologs that 82% of the genes predicted by Weiss et al. to be part of the LUCA genome are in fact false positives (Berkemer and McGlynn, 2020). Of particular interest is the protein, reverse gyrase, which is present in all hyperthermophiles. Catchpole and Forterre carried out an exhaustive phylogenetic analysis on reverse gyrase proteins, showing that tree topologies differed considerably from universal proteins inferred to be present in LUCA (Catchpole and Forterre, 2019). These results suggest that LUCA was not a thermophile, a conclusion that supports an earlier study on the evolution of thermophilic lifestyles after LUCA (Boussau et al., 2008). Numerous other studies attempt to define LUCA (Tuller et al., 2010; Lake et al., 2018; Koonin et al., 2020b) while a recent study even uses the current distribution of viruses across the ToL to reconstruct the types of viruses that infected LUCA—in other words, the LUCA virome (Krupovic et al., 2020). It is interesting to note that speculation exists as to whether the LUCA genome was made of DNA or RNA. Forterre builds on the hypothesis that cellular DNA originated from viral transfer by DNA viruses into RNA cells. He suggests the three domains of life each separately received their DNA genomes by three independent transfers, explaining the lack of homology between replicative DNA polymerases (DNAP) in Bacteria, Archaea, and Eukarya (Forterre, 2006). This hypothesis is intriguing because it posits a scenario where viruses are central to evolution at an early stage of the ToL—the origin of the three domains themselves. Koonin et al. think it more likely that LUCA had a DNA genome based on homology among different replicative DNAP and RNA polymerases (RNAP). In this scenario, RNAPs and DNAPs evolved from a common ancestor in an RNA-protein world that predated DNA replication, and LUCA’s DNAP is the ancestor of that existing in archaea today (PolD). While replicative DNAPs across the three domains are not homologous, Koonin et al. (2020b) point out that other aspects of the replication apparatus are universally conserved, suggesting that their common ancestral genes existed in LUCA.

**FIGURE 4:**
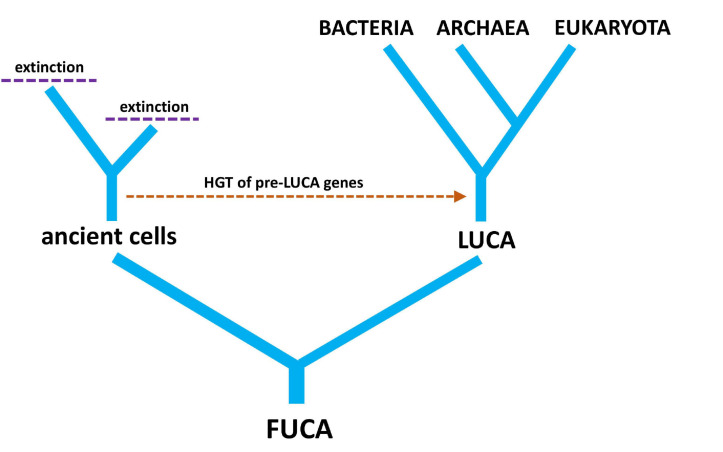
A simple tree diagram showing the relationship between LUCA (last universal common ancestor) and FUCA (first universal cellular ancestor). The hypothetical transfer of pre-LUCA genes from ancient cells into post-LUCA genomes is also depicted, along with extinction events of cellular lineages that have no modern descendants.

LUCA was not the first living cell but the last that served as an ancestor of all modern species. Many non-LUCA populations of cells existed at the same time as LUCA, but they failed to leave any modern descendants (Lake et al., 2018). It is interesting to speculate on the genetic diversity that was lost from the world due to extinction events. It is more interesting still to wonder at those lineages of cells that evolved alongside LUCA, possibly contributing genes through HGT before going extinct (Figure 4). Just because there is an unbroken history of cellular division linking LUCA to all modern life does not mean that pre-LUCA genes do not reside on post-LUCA genomes (de Farias et al., 2019). This brings us back to the distinction between the evolutionary history of genomes versus the evolutionary history of individual genes—two competing ideas in the light of HGT.

Can the ToL survive the genomic revolution? The species ToL is only a very crude approximation of the complexity of biological evolution at a molecular level. The ToL is also a dynamic model that has evolved conceptually to include a forest of gene trees, statistical central trends, and a network encapsulating both vertical and horizontal gene transfer. Such complex models are already digital in nature, being presented to us pictorially in studies for ease of understanding. How the earliest life began and subsequently evolved still holds mystery. Can viruses be placed within our tentative explanations of early biological evolution? As with cellular life they are also based on information carried in nucleic acids and they share the same or a very similar genetic code (Shackelton and Holmes, 2008). Comparative viral genomics is beginning to give us some answers (Bin Jang et al., 2019), while research into protein fold superfamilies (FSFs) shared by viruses and cellular lifeforms is suggestive of ancient homologies (Nasir and Caetano-Anollés, 2015).

Remembering again the online Open ToL (Hinchliff et al., 2015), is it possible to replace or even enhance a species-level focus with information about HGT and the plasticity of genomes? If viruses are truly inseparable from the evolutionary history of cellular life, how can we, in principle, deny them access to the ToL? A new framework for understanding the origin and evolution of viruses comes in the form of ancient, conserved protein structures (Koonin et al., 2020a), novel hypotheses about viral origins (Forterre, 2006; Forterre and Prangishvili, 2009; Kazlauskas et al., 2019; Krupovic et al., 2019; Nasir et al., 2020), and virus gene-sharing networks (Iranzo et al., 2016).

## The Origin and Evolution of Viruses

### Three Outdated Hypotheses About Viral Origins

Did viruses originate in a single event? Or did they arise independently on multiple occasions? Did they arise before or after the ancestor of all modern cells? Three general frameworks have been used to explain the origin of viruses, each of which was once thought to be mutually exclusive, while also leaving many unanswered questions (Nasir et al., 2012). In the virus-first hypothesis, the origin of viruses predated the origin of cells. These viruses would have arisen before cellular parasitism, perhaps existing as free-living replicators. How did they evolve to get inside cells and usurp their cellular machinery? Where did their capsids come from? The reduction hypothesis sees viruses evolving from cellular ancestors. In this scenario, viruses evolve after FUCA and before LUCA. The most popular scenario is one in which some lineages of cells have already evolved to parasitize other lineages, their genomes then shrinking over evolutionary time to a minimalist parasitic lifestyle (Nasir et al., 2012). This is how bacterial endosymbionts evolved their reduced genomes, such as many species of the genus *Mycoplasma* (Razin et al., 1998). We can envisage a parasitic genome losing genes to become more dependent on the host cell, but how would it then evolve to package itself inside a protein shell before bursting the cell open to spread out and infect new cells? The escape hypothesis posits the evolution of cellular genes that break away from the coordinated efforts of the genome to adopt a parasitic existence. This hypothesis is associated with the multiple and independent origin of viruses in all three domains of life—Bacteria, Archaea, and Eukarya—although there is evidence to suggest that monophyletic virus groups are not confined to a single domain (Iranzo et al., 2016). While this adaptive strategy can be linked to theoretical models on the emergence of parasitism (Koonin et al., 2017), the presence of genes that are unique to viruses suggests that not all viral genes originate from cellular homologs (Nasir et al., 2012). In fact, there is evidence to suggest that many novel genes originate in viruses and that gene flow between viruses and their hosts is dominated by host acquisition of viral genes (Forterre and Prangishvili, 2009). These three hypotheses on viral origins do not cover the full breadth of possibilities. They do, however, provide a good framework from which to interpret the outpouring of results from comparative genomic analyses that focus on ancient evolutionary events.

### Dispelling Viral Supergroups and the Fourth Domain of Life

Some viruses have only a handful of genes, while others have hundreds. DNA viruses generally have more genes than RNA viruses and, within each of these categories, ds viruses tend to have more genes than ss. Giant viruses containing thousands of genes were first discovered in 2003. They are the largest members of the phylum Nucleocytoviricota that multiply within molecular virus factories in the host cytoplasm and they primarily infect species of ameba. Their gene repertoire includes informational genes formerly thought to be exclusive to cells, a finding that led to a rethinking of the very notion of viruses (Brandes and Linial, 2019). Given what is known about HGT, it is sensible to ask if these informational genes were acquired from cellular hosts or have a more ancient origin, perhaps predating the common ancestor of all modern cells. The latter scenario promotes the virus-first hypothesis or, alternatively, the reduction hypothesis where giant viruses evolved to lose the full genetic toolkit required for independent existence, gradually adapting to a parasitic lifestyle. Virus-first pushes Nucleocytoviricota back to a pre-cellular origin, while reduction sees them evolving from a primitive cell that existed before LUCA (Moelling and Broecker, 2019).

Nucleocytoviricota were proposed as a fourth domain of life in 2010. A phylogenetic tree was built from a subset of informational genes, showing this group of viruses to be clearly distinct from Bacteria, Archaea, and Eukarya. Some translational genes were predicted to have been horizontally transferred from eukaryotes, suggesting a complex genetic history of ancient vertical transmission accompanied by HGT from other domains of life (Boyer et al., 2010). Forterre et al. argued that proposing a fourth domain of life from viruses ignores fundamental differences between viruses and cells. They suggest that the term “domain” should be restricted to descendants of LUCA based on ribosome structure and that viral evolutionary relationships should be ascertained by means of virion architectures and major capsid proteins (Forterre et al., 2014). The fourth domain hypothesis was later criticized for a failure to account for non-phylogenetic signals in the sequence data. Williams et al. used more realistic models of evolution to show that they could not reject horizontal acquisition of the same informational genes from eukaryotic hosts (Williams et al., 2011). This finding was later backed up by phylogenomic analyses showing giant viruses evolving multiple times from smaller Nucleocytoviricota ancestors. Gene gain from host genomes is therefore the recurring theme in this virus group, although “the evolutionary forces that led to the emergence of virus gigantism remain enigmatic” (Koonin and Yutin, 2019). While Nucleocytoviricota do not form a separate domain, recent evidence suggests they played an important role in the evolution of modern eukaryotes. They have been implicated in the origin of the eukaryotic nucleus, while phylogenetic analysis of informational proteins suggests that transfer took place between ancestral giant viruses and eukaryotes, possibly in both directions (Forterre and Gaïa, 2016). This idea is supported by a recent analysis of eight conserved proteins in Nucleocytoviricota that splits the phylum into two superclades and suggests that two transfers of DNA-dependent RNAP happened, one from each clade, from ancestral giant viruses to proto-eukaryotes (Guglielmini et al., 2019). These studies further highlight the influence of viruses on the evolution of cellular lineages.

The story of giant viruses is reminiscent of the difficulties of studying the ancient past by means of information rooted in the present. Inferring ancient evolutionary events from modern molecular data is like walking a tightrope, finding a balance between being too careful, and missing the opportunity to advance novel concepts. The dismissal of a viral supergroup tells a similar story. Protein FSFs are shared between viruses and cells, suggesting distant common ancestry. The abundance of these FSFs distributed across cellular lifeforms and the seven Baltimore viral classes was used in a phylogenomic exploration of viral origins and evolution. The results mistakenly suggested that all viruses originated as a supergroup from a primitive cell before the existence of the common ancestor of all modern cells. The study suggested that RNA viruses predated DNA viruses and evolved multiple times from ancient cells co-existing with LUCA, losing genes over evolutionary time (Nasir and Caetano-Anollés, 2015). This is an interesting concept that has been questioned by more recent analyses that highlight systemic errors biasing the outcome and interpretation of a viral supergroup. Harish et al. show that small-genome attraction artifacts as well as location of the root of the ToL distort these results into showing a common virus ancestor when, in fact, this is not well supported by the evidence (Harish et al., 2016). There is therefore no viral supergroup that originated as a monophyletic clade from primitive cells.

Likely, viruses did not evolve just once. Viral supergroups and extra domains present simplistic scenarios where viruses remain largely separated from the evolution of their cellular hosts. But viruses can be viewed more as a strategy and less as a single lineage that originated in a single time and place. They are more likely to have a multifaceted history, fully embraced by the concepts of the biological revolution brought about by genomics and HGT. Forterre writes that “the origin of viruses then becomes the question of the origin of virions as a specific mechanism of gene dissemination in the RNA/protein world” (Forterre, 2011). There is no reason to assume that a strategy as successful as virion production arose just once.

### Placing Viruses on the Tree of Life

**FIGURE 5:**
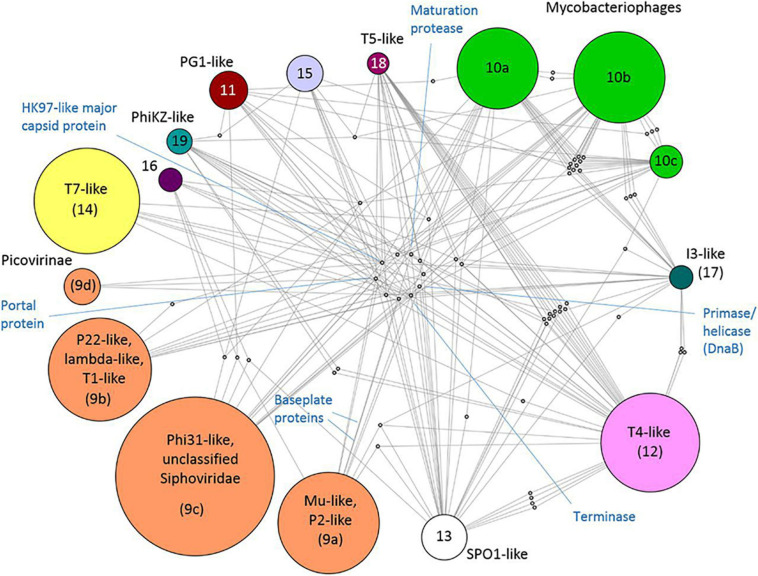
Taken from Iranzo et al. (2016). Internal structure of the *Caudovirales* supermodule. A bipartite graph is displayed, linking bacterial modules to the genes they share. Proteins that represent hub nodes in the gene-sharing network are labeled. License: CC Attribution 4.0 International License.

There is no universal gene that ties all viruses together in a phylogenetic framework. This was once possible only for closely related viruses, but virus gene-sharing networks have shown that the virosphere is more connected than previously thought. Iranzo et al. built a modular hierarchical network of gene sharing for the dsDNA virosphere. The network consisted of 19 modules, forming five major and three minor supermodules. Eleven of these modules included tailed bacteriophages (Caudovirales), highlighting the diversity of these viruses. They also discovered 14 viral hallmark genes (VHGs), which accounted for most of the inter-module connections. These hallmark genes included essential structural proteins and those involved in virus replication. Two major capsid proteins (double jelly roll and the HK97-like) acted as network hubs for the two largest supermodules: (1) HK97-like: tailed bacteriophages and herpesviruses (Figure 5) and (2) double jelly roll: the putative order Megavirales and smaller viruses, as well as polintons, which are large DNA transposons (Iranzo et al., 2016). In a separate study, Bin Jang et al. (2019) assigned many previously unclassified viruses to known viral genera using gene-sharing profiles and a network-based approach, showing that a large fraction of the viral sequence space remained unclassified. This finding is not surprising given the exponential increase in available virus genomes and the immense genetic diversity of the virosphere. It also agrees with Forterre and Prangishvili’s suggestion that we should abandon the “pick-pocket” hypothesis of viral gene origin, which views viruses as byproducts of evolution that capture genes from cellular lineages (Forterre and Prangishvili, 2009).

Viral hallmark genes tell us that viruses have a global organization, even if every species of virus cannot be brought together in a single phylogenetic model. A prime example of this is the RNA-dependent RNA polymerase (RdRp) involved in replication of RNA, which shares homology across dsRNA, +ssRNA, and -ssRNA viruses. More intriguing still is the presence of a so-called palm domain that is also found in the reverse transcriptase enzymes of RNA and DNA retroviruses. There is evidence that these enzymes form a monophyletic group, covering five of the seven Baltimore classes of viruses as well as group II introns, a large family of retroelements that multiply by splicing in and out of bacterial DNA (Koonin et al., 2020a). RdRp and RT have no cellular homologs apart from those that have been captured by cells from MGEs. The RNA viruses, including the two classes of retroviruses, have been elevated to a new taxonomic rank, the realm Riboviria, in a recently proposed megataxonomy of the virus world (Koonin et al., 2020a). Riboviria evokes the RNA world hypothesis, with RdRps and RTs possibly representing the modern descendants of pre-cellular RNA replicators. Alternatively, they might be the descendants of ancient cells that have no modern counterparts. Another enzyme, the rolling-circle replication endonuclease (RCRE) might have also come from an RNA world ancestor, having no cellular homologs (except for plasmids) and being found in both dsDNA and ssDNA viruses (Koonin et al., 2020a).

In this scenario of viral origins, RNA viruses predated LUCA. More specifically, the replication modules of RNA viruses predated LUCA, but this says nothing about their capsids and related structural proteins—the origin and evolution of viral capsids tells a different story. Numerous capsid-like structures are present in cells. A good example of these is bacterial microcompartments (BMCs). BMCs form shells that compartmentalize certain biochemical reactions in the cytoplasm. They are composed of two shell proteins, BMC-H and BMC-P, that form an icosahedral assembly bearing a striking morphological resemblance to viral capsids. The similarity ends there, however, as neither protein shares structural similarity with viral capsid proteins. Current evidence suggests a cellular origin of BMCs and, indeed, the recruitment by viruses of many cellular structural proteins (Krupovic and Koonin, 2017). Many +ssRNA viruses infecting eukaryotes have a single jelly roll capsid protein (SJR-CP). It is hypothesized, based on conserved protein structures, that the SJR-CP was derived from ancestral cellular carbohydrate- or nucleotide-binding proteins. The protein was co-opted by a parasitic RNA replicator that likely behaved much like plasmids or transposases do today. The combination of a replication module with a structural module gave rise to the first modern viruses. There is also evidence to suggest that the double jelly roll capsid protein (DJR-CP) evolved by gene duplication of the SJR-CP in an ancestral virus genome (Krupovic et al., 2019).

**FIGURE 6:**
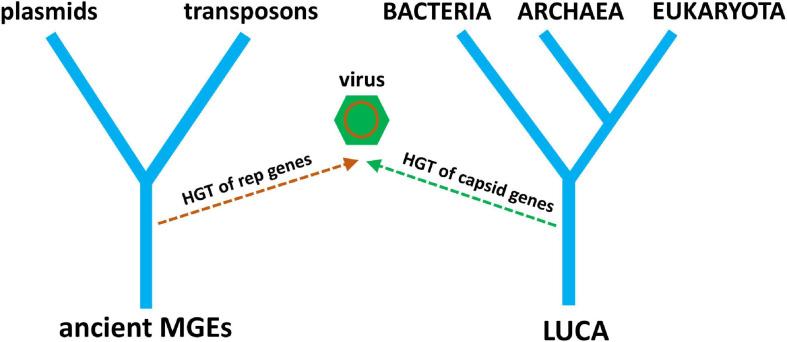
A simple tree diagram showing the chimeric origin of viruses from pre-LUCA replication genes and post-LUCA structural genes. Ancient MGE ancestors replace ancient cells (from Figure 4), reflecting the origin of virus replication genes from MGEs. The evolution of modern plasmids and transposons from ancient MGEs is also depicted.

It is becoming clear that the evolutionary histories of viruses and other MGEs are inseparable. It is also clear that cellular life has not evolved separately from the genetic parasites that have evolved to exploit it. The origin of RNA viruses is currently explained by a hybrid of two hypotheses, virus-first and escape, where the replication module has a virus-first origin and the structural module has an escape origin (Figure 6). What about the origin of DNA viruses? Do they tell a similar story of conflicting evolutionary histories? Nowhere is this more revealing than for the multiple, chimeric origins of ssDNA viruses. The RCRE enzyme hypothesized to be part of the RNA world existed as a bacterial plasmid before it existed as ssDNA viruses. Three lineages of ssDNA viruses—inoviruses, pleolipoviruses, and microviruses—evolved independently from RCRE plasmids by co-opting a filamentous, polymorphic, and SJR capsid protein, respectively. The eukaryotic Rep-encoding ssDNA viruses (CRESS-DNA) evolved when a superfamily 3 helicase (S3H) was incorporated into an RCRE plasmid, followed by several co-options of non-homologous SJR. Amazingly, these capsids come from diverse +ssRNA viruses infecting animals and plants, recombining on multiple, independent occasions with RCRE-S3H plasmids in a remarkable display of convergent evolution (Kazlauskas et al., 2019). A virus with plasmid origins, coupled with the co-option of existing viral capsids from a different type of replicator is a fascinating scenario. A plasmid ancestor begs an obvious question: if there is a place for viruses on the ToL, why not plasmids too? We can logically ask the same question about other selfish replicators such as DNA transposases, which, after all, show distant homology to viral sequences (Iranzo et al., 2016).

Single-stranded DNA viruses have recently been given a realm of their own: Monodnaviria. This taxonomy is sensible because ssDNA viruses all evolved from the same type of RCRE plasmid. The origin of dsDNA viruses is far less certain than that of ssDNA, but there are two major divisions defined by the presence of either a DJR or a HK97-like major capsid protein. Despite a highly variable number of genes, and evidence of HGT between the two supergroups, they have been split into two realms, Varidnaviria and Duplodnaviria, suggesting an ancient and independent origin for both realms (Koonin et al., 2020a). RNA replication and reverse transcription are unique to viruses and MGEs, except for cases of co-option and subsequent adaptation into cellular processes (Koonin et al., 2020a). It is therefore likely that these types of replication existed before LUCA, back in the primordial world. While ssDNA viruses arose more recently, dsDNA may also have existed alongside RNA replicators, perhaps competing as an alternative replication strategy on a young Earth. It is interesting to note that two dsDNA virus groups, papillomaviruses and polyomaviruses, originated from a ssDNA ancestor (Kazlauskas et al., 2019).

It appears that the viral strategy of genome propagation is an ongoing experiment among biological entities. Forterre states that “the tree of life is infected by viruses from the root to the leaves” (Forterre et al., 2014). This metaphor captures the numerous, independent origins of viruses from early RNA parasites pre-dating LUCA to more recent viral lineages such as the ssDNA viruses that evolved from plasmids combined with RNA capsid proteins. It is obvious that there is no single branch into which viruses can be placed. It is likely that many viruses are a hybrid of genes from divergent lineages, existing both before and after the emergence of LUCA (Koonin et al., 2020a). Viruses also played major roles in the origin and evolution of numerous cellular lineages, perhaps even driving the emergence of the three cellular domains of life (Forterre, 2006). Where does this leave us with viruses and their place on the ToL? Accepting the ToL as a dynamic model of the evolution of biological entities on Earth, viruses should rightly be included in these models. The question then becomes not if viruses have a place on the ToL, but how and where should they be placed? It is a difficult task, however, to put these principles into practice.

Koonin et al. (2020a) state that it is likely “a comprehensive account of virus evolution can be achieved only through the combination of phylogenetic and network approaches.” It is also clear that virus evolution is inseparable from evolution of cellular lineages. There is an inherent tree-like nature to replicating sequences and Puigbò et al. (2013) have described a forest of gene trees as a more natural representation of the biological evolution of replicators. This view is also supported by Forterre (2012) who reinforces this concept by writing that “*[a]s soon as an object divides by duplication, the history of that object has a tree-like structure*.” Gene-sharing networks describe sequence homology in cases where clear tree-like patterns of evolution are difficult or impossible to decipher. Forterre (2012) goes on to say that “we should not try to escape these difficulties by replacing trees with networks” and Koonin et al. (2009a) also offer the opinion, referring to the FoL, that “evolutionary trees of viral genes legitimately belong in that forest.” This is especially true since viruses are being shown more and more to be the cradle of new genes (Forterre and Gaïa, 2016), so many of these trees in the FoL are of viral origin.

**FIGURE 7:**
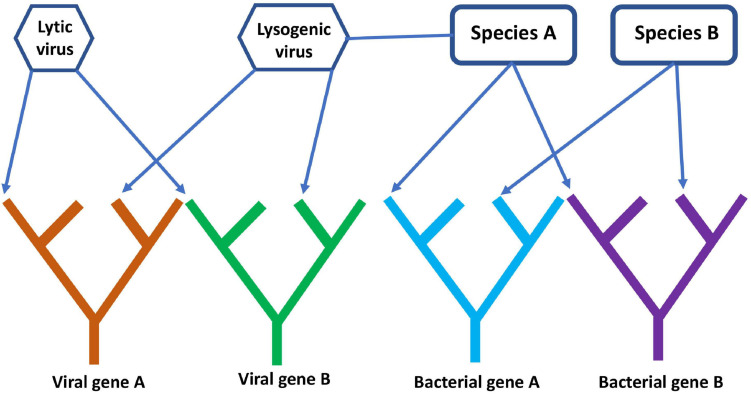
A simple diagram of the phylogenetic forest of gene trees with network components linking different genes together. The diagram shows four gene lineages, two viral and two cellular. Arrows connect genes present on the same genome to their respective viral or bacterial species, while the lysogenic virus is connected to species A, showing that it is a prophage whose genes reside on the same genome as species A.

A comprehensive, digital representation of all these trees removes the network component because the evolutionary history of each gene is treated separately. This is somewhat simplistic since genes can gain and lose domains over time (Nasir et al., 2014), so individual gene trees can also become intertwined unless domains are also represented by separate trees. This gene’s eye view of molecular evolution is very different from the species ToL that existed in Darwin’s era, but an FoL is closer to the biological reality of what has occurred since evolution began. Separate gene trees hardly paint a complete picture of evolution though since genes interact and often replicate together within cells. A network structure could still be added to the FoL by connecting individual gene trees or specific gene tips to each other to represent higher level organization at the genome, organismal, or species level. In principle, such a digital forest with network components could record both the vertical transmission of genes within lineages and the horizontal transmission of genes across lineages. For example, the lytic and lysogenic lifecycles of viruses and their association with host species and genomes could be represented (Figure 7). It is important to note that models are only useful if they can be used to answer questions. For many purposes, the standard species ToL might be perfectly sufficient. For understanding the co-evolution of viruses and other MGEs with cellular life, such a tree is inadequate.

Genetic parasites are an inevitable outcome of replicator systems (Koonin et al., 2017), reflecting perhaps the most fundamental of strategies after replication itself. Conserved, ancient structural proteins have revealed an entangled evolutionary history of all MGEs. Krupovic et al. (2019) summarize their hypotheses on the origin of viruses by concluding that “[t]he tight evolutionary link between viruses and capsidless MGEs is the core of our model of the origin of viruses.” Meanwhile, Gill et al. (2019) describe the connection between viruses and extracellular vesicles today, hypothesizing about the potentially important role of EVs in the origin and evolution of the first viruses. Whatever the details of the multiple origins and evolutions of viruses, there is no reason to exclude them from our models of biological evolution on Earth. The difficult part will be to build these models to competently represent the origin and co-evolution of viruses with cellular life.

## Concluding Remarks

Life is the outcome of billions of years of experimentation on a planetary scale, with processes that we are only beginning to fathom, and the outcomes appear to be an almost unlimited number of dynamic strategies for replicators to exist and multiply in the world. We have discovered the importance of HGT in genome evolution only relatively recently, yet its molecular basis likely predated the evolution of cooperation. Genetic cooperation and genome organization were therefore preceded by selfishly splicing replicators (Dawkins, 1976). It is also possible, although speculative, that all modern MGEs are descendants of ancient replicators that existed before cooperative behavior.

The scientific community will never fully agree on the living nature of viruses and other MGEs. Opinions range from Moreira and López-García (2009) who state that viruses are not alive to Forterre (2016) who posits that mitochondria, viruses, and even proteins can be considered living once they are functional within living systems. We favor an open-minded view in this article, but we think the living nature of viruses does not ultimately matter as much as the fact that they are evolving biological entities that have co-evolved with cellular life and engaged in regular HGT with their hosts, likely playing pivotal roles in cellular evolution.

Our understanding of life is limited but growing. We need dynamic and evolving models that can answer our questions about the nature of biology. In this review, we argue that viruses should be included in future models of biological evolution—models that have historically been represented by the ToL. These models will need to be digital and multi-dimensional in nature. They will also be very difficult to create. One option is to be cynical and heed the words of Steven Benner on lifeforms: “We do what we generally do when a reality is too complex to meet our constructive needs: we ignore it and continue with a simpler, if arguably false, view” (Benner, 2010). But every model of reality is necessarily a construct. Our brains themselves are comprised of cooperating and competing neuronal modules and sub-modules (Rutishauser et al., 2018); it is extraordinary that our understanding has got this far.

What we know from the scientific method is that some views are less false than others. Koonin et al. (2020a) conclude on a positive note that a “comprehensive, internally consistent, and stable hierarchical taxonomy of viruses seems to be within the reach of the current generation of virologists.” We hope that this timeframe might be true, as well, for understanding the origin and evolution of viruses in the living world.

## Author Contributions

Both authors conceived of the review topic and layout, wrote the final draft of the manuscript, and approved the final version. HH wrote a complete draft of the manuscript.

## Conflict of Interest

The authors declare that the research was conducted in the absence of any commercial or financial relationships that could be construed as a potential conflict of interest.
